# Reducing Postpartum Weight Retention: A Review of the Implementation Challenges of Postpartum Lifestyle Interventions

**DOI:** 10.3390/jcm10091891

**Published:** 2021-04-27

**Authors:** Maureen Makama, Helen Skouteris, Lisa J. Moran, Siew Lim

**Affiliations:** 1 Monash Centre for Health Research and Implementation, Monash University, Clayton, VIC 3168, Australia; maureen.makama@monash.edu; 2 Health and Social Care Unit, School of Public Health and Preventive Medicine, Monash University, Melbourne, VIC 3004, Australia; helen.skouteris@monash.edu

**Keywords:** postpartum weight retention, postpartum lifestyle interventions, intervention components, implementation

## Abstract

Postpartum weight retention (PPWR) is a strong predictor of obesity in later life with long term health consequences in women. Suboptimal lifestyle behaviours (e.g., diet and physical activity) contribute to PPWR. Postpartum lifestyle interventions are known to be efficacious in reducing PPWR; however, there are challenges to their successful implementation. To inform implementation, this narrative review provides an overview of the factors that contribute to PPWR, the efficacy of existing postpartum lifestyle interventions and key determinants of effective implementation using the Consolidated Framework for Implementation Research (CFIR) across intervention characteristics, implementation process, individual characteristics and outer and inner setting. We then suggest strategies to improve the translation of evidence into large-scale interventions that deliver on health impact in postpartum women. We have identified gaps that need to be addressed to advance postpartum lifestyle research, including the involvement of postpartum women and community members as key stakeholders for optimal reach and engagement, more complete reporting of intervention characteristics to optimize translation of evidence into practice, capacity building of health professionals and guidelines for postpartum lifestyle management.

## 1. Introduction

The prevalence of overweight and obesity is on the rise globally, presenting a challenge to public health [1]. In 2016, 44% (>2 billion) of adults worldwide had overweight or obesity [1]. In particular, women of reproductive age are at an increased risk of having overweight or obesity [2]. Global data reports that among women, the prevalence of having overweight and obesity was 39% and 15% respectively in 2016, compared to around 23% and 6%, respectively, in 1975 [3]. Weight gain associated with childbirth contributes to obesity risks in women [2,4], with excessive gestational weight gain (weight gain above the Institute of Medicine (IOM) recommendations during pregnancy) and postpartum weight retention (PPWR) (retention of the weight gained during pregnancy) being significant contributors to parity-related weight gain [5,6]. Excessive gestational weight gain is also a major predictor of PPWR both in the short and long term [5,6,7,8,9,10]. On average PPWR ranges from 0.5 to 3 kg; however, this is highly variable, with up to 20% of women retaining >4 kg at 1 year postpartum [5,6,11]. Weight retention after the first postpartum year is associated with weight retention up to 15 years later [12]. PPWR is therefore a strong predictor of obesity in later life and also predisposes women to an increased risk of chronic diseases such as cardiovascular disease, diabetes, osteoarthritis and some cancers [6,7,11,12]. This constitutes a significant economic burden in terms of increased healthcare costs, reduced productivity, increased disabilities and reduced length of disability-free healthy living across the life cycle [1,13]. Furthermore, PPWR contributes to adverse outcomes in subsequent pregnancies such as preeclampsia, gestational diabetes mellitus, caesarean section, stillbirth, congenital anomalies, increased birth weight and being born large for gestational age [14,15].

A range of factors contribute to PPWR including lifestyle behaviours, sociodemographic and psychosocial factors. An understanding of these could help inform intervention development to address PPWR. Postpartum lifestyle interventions to date targeted mainly lifestyle behaviours without addressing other contributing factors. Despite these limitations, these postpartum lifestyle interventions have demonstrated efficacy in weight and lifestyle management as shown in previous meta-analyses [16,17]. However, evidence on efficacious interventions from nearly three decades has not been effectively translated to changes in practice or policies [18]. Most maternal health guidelines around the world do not address postpartum weight or lifestyle [19]. There is need for clear implementation strategies to guide evidence translation into practice to deliver public health impact.

This narrative review aims to inform implementation of postpartum lifestyle interventions in clinical practice. First, we provide an overview of the factors that contribute to PPWR and the efficacy of existing postpartum lifestyle interventions. Then, using the Consolidated Framework for Implementation Research (CFIR), we summarize the key determinants of effective implementation of postpartum lifestyle interventions across intervention characteristics, implementation process, individual characteristics and outer and inner setting. The CFIR is a comprehensive implementation science framework that guides the identification of factors that may influence implementation outcomes [20,21]. It incorporates constructs from 19 implementation theories, frameworks and models into a single comprehensive framework [20]. CFIR was selected to guide this review because it provides an overarching perspective on implementation strategies necessary to ensure improvement in adoption, implementation, sustainment and scale-up of postpartum lifestyle interventions [22]. Finally, we summarize the key findings for the implementation of postpartum lifestyle interventions according to CFIR and provide recommendations for research and practice.

The literature search was conducted in November 2020 using PubMED and Google Scholar databases. Key search terms were “postpartum AND postpartum weight retention AND (postpartum lifestyle intervention OR diet OR nutrition OR physical activity OR exercise OR weight management OR sedentary behaviour OR depression OR anxiety OR stress OR sleep OR psychological factors OR breastfeeding)”. We included only systematic reviews of primary studies that examined the predictors of or factors associated with postpartum weight retention and postpartum lifestyle interventions with weight-related outcomes. To be included, interventions had to have occurred in the postpartum period, including those commencing during pregnancy. Only reviews published in English were included without limitation to the year of publication. The quality of the included studies was assessed using AMSTAR 2, a measurement tool to assess systematic reviews [23].

## 2. Predictors of Postpartum Weight Retention

Lifestyle behaviours, sociodemographic and psychosocial factors may contribute to PPWR [24]. Sociodemographic factors known to be associated with PPWR include maternal age of under 20 or over 40 years, primiparity, certain ethnicity (e.g., African), unemployment, low income and low education level [24,25,26,27]. Smoking cessation during pregnancy or early postpartum is also associated with long-term weight gain [11,28]. 

There is generally a decline in healthy dietary behaviours from pregnancy through to postpartum [29,30,31] (Table 1). Diet quality was reported to be higher in the first postpartum year compared to subsequent years indicating that the decline in diet quality continues even after the first postpartum year [31]. Lee et al.’s review reported less adherence to healthier dietary patterns, specifically decreased fruit and vegetable intake and increased intake of energy dense and nutrient poor food during the transition from pregnancy to postpartum [30]. This worsening of lifestyle behaviours in the postpartum period could be due to the demands associated with caring for a child [32]. There is inconsistency in the literature on the association between diet quality and PPWR. While several studies report no association [33,34,35], a recent study reported that lower diet quality was independently associated with PPWR [36]. Furthermore, total energy intake irrespective of diet composition and the consumption of trans fat and discretionary foods have been reported to be associated with PPWR [33,36,37,38].

Irregular sleep and meal times during the postpartum period could also interfere with body weight. Emerging evidence suggests that the misalignment of eating and fasting patterns with the circadian rhythm of the body could impact on metabolic function and consequently body weight [47]. A recent study reported that greater caloric intake at night was independently associated with PPWR [36]. A positive association between short sleep duration and PPWR has also been reported in a previous review [42]. In primary research, short sleep duration of ≤5 h daily in the first postpartum year was associated with more PPWR [24,48,49].

Physical activity levels are generally inadequate in postpartum women [50]. The decline commences in late pregnancy and although there is a progressive improvement 3 to 12 months postpartum, it still remains lower than pre-pregnancy levels [51,52,53,54,55,56]. The decline in physical activity is observed in leisure time physical activity [54] and moderate to vigorous physical activity but not in walking [55,56]. Postpartum physical activity is beneficial for improvement of aerobic fitness, insulin sensitivity and psychological wellbeing [57,58,59]. Physical activity may also be beneficial for postpartum weight loss [60,61]; however, the evidence on its effect is limited [57,58,59]. In the general population, physical activity has an additive effect when combined with dietary restriction on weight loss but in isolation has only a modest effect, requiring relatively high doses to enhance long-term weight loss and minimize weight regain [62]. In postpartum women, the maintenance of light physical activity such as walking, with a decline in moderate to vigorous physical activity may be insufficient by itself for weight control [55,56]. Therefore, physical activity may need to be combined with dietary interventions to obtain maximum benefits in weight management, as per the general population [62].

Sedentary behaviours may also be associated with PPWR. Oken et al. reported that women who watched fewer than two hours of television daily were less likely to retain ≥5 kg postpartum at 1 year postpartum [37]. However, literature on the association of sedentary behaviour with PPWR is sparse. Limited primary research has reported a decline in sedentary behaviour in postpartum women compared to during pregnancy [53,55].

Psychosocial factors such as depression and anxiety are also predictors of PPWR (Table 1). Previous systematic reviews reported the prevalence of depression and anxiety in the postpartum period as 17% and 15%, respectively [63,64]. A positive association between postpartum depression and PPWR or maternal obesity [40,42,43,65] but no association between anxiety [40,41] or stress and PPWR [42] were reported in previous reviews. Several individual studies suggest a positive association of depression and life stress with PPWR [66,67]. A large prospective cohort study reported that women who reported feeling depressed or anxious during pregnancy and in the first 6 months postpartum had the highest PPWR at 18 months postpartum [26]. Dealing with stress associated with infant hospitalization has also been reported to be associated with PPWR [24]. Eating to cope with psychological, emotional and physical stress may explain some of the association of psychosocial factors with PPWR [68]. A previous review found an inverse relationship between healthy dietary patterns and perinatal anxiety and depression, suggesting that the association of psychosocial factors with PPWR may be linked to eating behaviour [41,69].

There is inconsistency in the literature on the effect of breastfeeding on postpartum weight with systematic reviews reporting beneficial [44], negative [45] or inconclusive [46] effects of breastfeeding on PPWR (Table 1). Jiang et al. reported a beneficial effect of breastfeeding for 6–12 months which was more pronounced in women younger than 30 years old, primipara or having normal pre-pregnancy BMI [44], while He et al. reported a negative effect of breastfeeding ≤6 months and no effect of breastfeeding >6 months [45,46]. Neville et al.’s systematic review concluded that there was insufficient evidence to suggest that breastfeeding was directly associated with postpartum weight change [46] with 63% of the observational studies included in the review reporting no significant differences in postpartum weight change between breastfeeding and non-breastfeeding mothers [46]. However, four out of five studies deemed to be of high methodological quality demonstrated a positive association of continuous breastfeeding up to 12 months and longer duration of exclusive breastfeeding with postpartum weight change [46]. The challenges to the evidence synthesis of the impact of breastfeeding on postpartum weight include inconsistency in definitions used across studies, difficulty in quantifying the duration and intensity of breastfeeding and inadequate adjustment for potential confounders of the association [11,45,46]. The energy cost of breastfeeding is up to 500 kcal per day, and therefore, lactation may help mobilize fat stores built up during pregnancy leading to weight loss provided there is no compensatory increase in energy intake [70]. The provision of breastfeeding support is a potentially effective strategy that has not been explored in postpartum lifestyle interventions for weight management [71], (Box 1).

Box 1 **Key findings on predictors of postpartum weight retention.**

- There is a decline in healthy dietary behaviours in the postpartum period
- There is a maintenance of light physical activity such as walking and a decline in moderate to vigorous physical activity in the postpartum period
- Depression is positively associated with postpartum weight retention but not anxiety and stress
- Short sleep duration is positively associated with postpartum weight retention
- There is inconsistency in literature on the association of breastfeeding with postpartum weight retention

## 3. Efficacy of Lifestyle Interventions in Postpartum Women

Diet and physical activity behaviours are modifiable lifestyle behaviours that can be targeted for postpartum weight loss. Several systematic reviews have explored the efficacy of lifestyle interventions for postpartum weight loss [16,17,71,72,73,74,75,76,77,78,79,80,81,82] (Table 2). Postpartum interventions including a combination of diet and physical activity components are efficacious for postpartum weight loss and improvement of body composition [17,81] with mean difference (MD) in body weight of −2.33 kg (95% confidence interval (CI), −3.10 to −1.56) reported [17] and sustained at 12 months postpartum [74]. Postpartum lifestyle interventions have also been reported to improve physical activity (standardized MD of 0.61, 95% CI (0.20 to 1.02)), but no significant effect on energy intake was reported [83,84,85]. While few systematic reviews have examined the efficacy of diet-only interventions, these report that they are also efficacious for postpartum weight loss [72,81]. However, physical activity/exercise-only interventions have been reported to be inefficacious for postpartum weight loss [72,75,81,86]. Despite the absence of significant weight loss [58,72,75], meta-analyses of exercise-only interventions reported improvements in maternal cardiovascular fitness [72] and postnatal depression [58], (Box 2).

Box 2 **Key findings on efficacy of lifestyle interventions in postpartum women.**

- Lifestyle interventions combining diet and physical activity components are efficacious for postpartum weight loss
- Diet-only interventions are efficacious for postpartum weight loss
- Physical activity only interventions are inefficacious for postpartum weight loss

## 4. Implementation of Postpartum Lifestyle Interventions: Identification of Core and Adaptable Components

Implementation is critical to the effective translation of existing evidence on the efficacy of postpartum lifestyle interventions into practice and to allow delivery of sustainable health impact on a large scale. Identification of both the core and adaptable components for intervention effectiveness as described in the CFIR is an important step in the implementation of postpartum lifestyle interventions [20]. The CFIR describes the major domains necessary to promote implementation across multiple contexts. These domains—intervention characteristics, inner and outer setting, individual characteristics and process of implementation are interrelated and interact in a complex way to influence implementation [20]. Below, we provide an overview of factors that may influence implementation of postpartum lifestyle interventions guided by the CFIR domains.

### 4.1. Intervention Characteristics

#### 4.1.1. Considerations for Program Delivery: Intervention Characteristics by TiDieR Framework

Intervention characteristics refer to what the content of interventions are, why they are effective, how they are delivered, by whom, at what frequency, for what duration, in what setting and how well they are delivered to be most effective. The intervention characteristics associated with weight loss in postpartum women have previously been identified using the Template for Intervention Description and Replication (TiDieR) framework [79] (Table 2). The TiDieR is a checklist and guideline developed to improve completeness in the reporting and the replicability of interventions [92]. The checklist consists of intervention characteristics such as the theoretical framework, materials, procedures, intervention delivery, frequency, duration and other determinants of the effects of interventions. Lim et al. reported that postpartum weight management interventions had greater efficacy when delivered by health professionals and when combining diet and physical activity components [79] (Table 2). This reiterates previous findings that physical activity needs to be combined with dietary interventions to maximize its benefits in effective interventions [71], (Box 3).

Box 3 **Key findings on intervention characteristics.**

- Efficacious postpartum interventions should include dietary and physical activity components and be delivered by health professionals
- Non-core intervention components that could be left to contextual needs of the target population include intervention intensity, duration, length, delivery mode and setting
- More research is required to determine the optimal timing for the initiation of postpartum lifestyle interventions
- The total number of BCTs included and including the BCTs ‘goal setting’ and ‘self-monitoring of behaviour’ are associated with greater reduction in energy intake and improvement of physical activity

There is no consensus on the optimal timing for the initiation of lifestyle interventions in postpartum women with timing ranging widely across studies from immediately post-birth to 18 months postpartum [16,80,82]. Previous systematic reviews of lifestyle interventions in postpartum women report intervention duration ranging widely from 3 weeks to 15 months [16,71,79]. Successful weight loss interventions were more likely to be of shorter duration of ≤6 months than of >6 months duration [16,71,79,90]. Lim et al. reported that other intervention characteristics such as duration and intensity did not have a significant effect on efficacy and may be adaptable based on contextual needs [79].

Previous interventions successful in promoting weight loss, dietary and physical activity behaviours have used a range of settings, including home-based and/or hospital clinic based [82,93]. The choice of intervention setting is critical considering that postpartum women face challenges of childcare and time constraints which may impact on their participation and adherence to lifestyle interventions [90,94]. Home-based interventions using telephone, internet or mail may be more practical for postpartum women than clinic-based interventions unless when interventions are embedded into routine visits to child health clinics [90,95]. The use of electronic health (e-health) technology has been reported to be perceived as a positive, user friendly and acceptable delivery medium for lifestyle interventions by postpartum women [96,97,98,99]. However, even primarily home-based interventions can have very low recruitment rates as noted by Jones et al.’s review with all interventions using primarily telephone or mailing reporting poor recruitment and participation rates (as low as 17%) [100,101,102], thereby highlighting the challenges of reaching postpartum women [93]. Individual versus group delivery did not significantly impact the efficacy of postpartum weight loss interventions in previous reviews [79,90]. Individual interventions allow for individualized feedback, while group-based interventions may be beneficial for social support [90,103]. Individually tailored counselling, group counselling sessions, and use of diaries or other correspondence materials have been reported to be effective [77]. The ideal setting and delivery format of postpartum lifestyle interventions should therefore seek to address the barriers of childcare, time constraints and social support in order to maximize engagement while tailoring to the contextual needs of the target population.

#### 4.1.2. Considerations for Program Content: Behaviour Change Techniques (BCTs)

Behaviour change techniques (BCTs) are the active ingredients of interventions aimed at behaviour change which can impact on the effectiveness of the intervention [104,105]. Identifying the most effective BCTs is therefore critical to successful behaviour change in a lifestyle intervention. Previous reviews have examined the most effective BCTs for improving weight, dietary and physical activity behaviours in postpartum women [83,85]. They reported that the total number of BCTs and including the BCTs ‘goal setting’ and ‘self-monitoring of behaviour’ were associated with greater reduction in energy intake and greater efficacy of physical activity interventions [83,85]. Additionally, Lim et al. identified that the provision of BCTs ‘problem solving’, ‘goal setting of outcome’, ‘reviewing outcome goal’, ‘feedback on behaviour’, ‘self-monitoring of behaviour’, ‘behavioural substitution’ and ‘credible source’ was associated with greater reduction in energy intake (Table 2). On the other hand, another review focusing on physical activity interventions reported that the BCTs ‘provide information on consequences of the behaviour in general’, ‘provide information on where and when to perform the behaviour’, ‘provide instruction on how to perform the behaviour’ and ‘barrier identification/problem solving’ were more likely to have been included in non-efficacious physical activity interventions compared to efficacious interventions. Self-monitoring has also been identified in other reviews as an important component of lifestyle interventions in postpartum women [71,76,82,90].

### 4.2. Implementation Process

#### Considerations for Population Reach: Penetration and Participation Rates

Although the efficacy of lifestyle interventions in postpartum women has been established, their impact at the population level is determined by the program reach (penetration) and engagement (participation) [106,107]. Therefore, for effective implementation and translation of postpartum lifestyle intervention programs, it is important to understand what strategies work best to reach and engage a significant proportion of the target population [17]. The impact of lifestyle interventions in postpartum women has previously been evaluated in a systematic review using the penetration, implementation, participation and effectiveness (PIPE) Impact Metric [17] (Table 2). Penetration refers to the proportion of the target population that is reached by invitations to engage in the program. Implementation is the fidelity of the intervention protocol, which is the degree to which the program has been implemented according to the design specifications. Participation refers to the proportion of invited target population who enrol in the program. Effectiveness is the measure of success in the participants based on the primary outcome [106]. The impact of a program on a population is a function of all these factors. Lifestyle interventions targeting postpartum women rarely address penetration, implementation and participation, focusing instead only on the intervention effects [17]. This leads to a lack of data to inform intervention strategies that are associated with better penetration or participation. This may contribute to the lack of translation from efficacy studies to real-world solutions in postpartum lifestyle management. A review by Dasgupta et al. also found suboptimal reporting of penetration and participation in interventions to prevent diabetes in the subpopulation of postpartum women of post gestational diabetes [108]. The few studies with high penetration or participation rate were embedded in existing services used by postpartum women such as parent groups, or started recruitment in pregnancy [108], (Box 4).

Box 4 **Key findings on implementation process.**

- Penetration and participation are rarely reported in randomized controlled trials and needs to be the focus of future research
- Studies with good penetration and participation rates were conducted within existing health services for postpartum women or involved recruitment during pregnancy

### 4.3. Individual Characteristics

#### Barriers to Lifestyle Management in Postpartum Women

Apart from including the core components identified in the previous section, it is important that interventions for postpartum women address the specific barriers to lifestyle management in this group. These barriers could hinder participation in lifestyle interventions and contribute to high attrition rates of up to 42% as reported in previous systematic reviews [16,78,90,109]. These barriers were summarized in a recent systematic review using the Capability, Opportunity, Motivation and Behaviour (COM-B) model, which is a behaviour change framework [94]. In terms of capability, barriers reported include limited knowledge on how to safely resume exercise after birth, fatigue, tiredness, sleep disturbances, stress and depression. In terms of opportunity, time constraints, prioritizing care for the child and household commitments over personal health, lack of access to childcare, financial constraints and lack of suitable environment for exercise are barriers women face. Lack of support from partners for healthy eating, exercising and childcare, from friends for exercise, and lack of lifestyle support from healthcare providers were also reported. In terms of motivation, the perception that great effort is required, lack of confidence in their ability to exercise, unwillingness to change eating habits, lack of enjoyment of exercise or healthy food and low self-worth were reported barriers to healthy lifestyle behaviours in postpartum women [94]. These barriers may explain poor attendance at interventions targeted at postpartum women as reported in previous studies [95]. Interventions targeting postpartum women should be tailored to address the unique barriers in this life-stage in order to improve engagement. Improving engagement may result in greater intervention effect as demonstrated in a postpartum lifestyle intervention aiming at reducing diabetes risk in women with previous gestational diabetes [103]. This study saw a greater reduction in weight and waist circumference when the delivery mode was changed from in-person group-delivered to telephone-delivered [103]. This led to an increase in engagement from 38% to 82% from addressing barriers of childcare, scheduling and accessibility [103]. Hartman et al. similarly reported that effective interventions included components targeted at mother-specific barriers [91], (Box 5).

Box 5 **Key findings on individual characteristics.**

- Postpartum women face barriers unique to this life-stage relating to capability (e.g. tiredness and fatigue), opportunity (e.g. lack of childcare and social support) and motivation (lack of confidence in their ability and lack of enjoyment of exercise)
- These barriers may result in low levels of engagement in lifestyle interventions

### 4.4. Outer and Inner Setting

#### 4.4.1. Societal Drivers and Culture

Determinants that influence engagement with lifestyle interventions beyond the individual woman include family and friends, cultural influence, health professionals, the built environment and socio-economic impact [110]. The influence of the family on postpartum women’s willingness to engage in lifestyle modification has previously been reported [84,94,111]. The support and involvement of partners in postpartum lifestyle interventions is particularly crucial for lasting behaviour change especially in high-risk women who have experienced gestational diabetes or preeclampsia [108,111,112,113,114]. Cultural values and norms can also impact on postpartum lifestyle choices [94].

#### 4.4.2. System Barriers

Support from health professionals play a key role in facilitating behaviour change in postpartum women. During pregnancy, women have regular contact with health professionals about both their health and the health of their foetus. However, health professional support in postpartum care is less regular and usually focused on breastfeeding and care of the child rather than on maternal health [32,35,115]. There is also a lack of clarity around the role and responsibility among health professionals on who is responsible for postpartum women’s health. Despite this gap in healthcare structure, the importance of health professionals’ involvement in the provision of care and healthy lifestyle support to women beyond pregnancy is clearly highlighted in previous systematic reviews reporting greater weight loss in postpartum interventions delivered by health professionals than those delivered by non-health professionals [71,79,82] (Table 2). Health professionals are valued as a credible source of information for the new mother; therefore, health professionals should use this opportunity to not only provide support in terms of breastfeeding and care of the newborn but also for lifestyle behaviour counselling and support. Makama et al. reported that health professionals themselves face barriers to the provision of lifestyle support to postpartum women including time constraints during consultations and limited lifestyle counselling skills [94]. There is therefore a need to train health professionals involved in the provision of care to postpartum women to improve their knowledge, skills and confidence in providing lifestyle management.

Comprehensive clinical guidelines for routine postpartum care are limited and do not include recommendations for lifestyle or behavioural counselling [116,117]. Guidelines for weight management in the postpartum period are similarly limited with a general lack of guidance on how to implement the evidence into clinical practice [19,118,119]. Recommendations on the timing and frequency of postpartum visits are variable and based on weak evidence [117]. There is a need for health care policies to incorporate lifestyle counselling into routine care for postpartum women. This could be through the integration of postpartum care with child immunization clinics. Financing postpartum care services could potentially serve as a barrier to its utilization; therefore, to ensure accessibility, health insurance coverage needs to be extended to these services. [120]. Optimizing postpartum care therefore requires policy changes [121], (Box 6).

Box 6 **Key findings on outer and inner setting.**

- Health professionals play a significant role in providing lifestyle behaviour counselling and support for postpartum women
- Health professionals face barriers of limited time and skills to provide support to postpartum women
- Capacity building of health professionals is required to ensure they are equipped to provide lifestyle support for postpartum women
- Clinical guidelines for postpartum care do not include lifestyle or behavioural counselling
- There is need for more research into the timing and frequency of postpartum visits

## 5. Recommendations for Research and Practice

The core components associated with efficacy of postpartum lifestyle interventions are: inclusion of both diet and physical activity components, delivery by healthcare professionals, use of electronic health technology, including more BCTs (especially self-monitoring and goal setting) and embedding interventions in existing services. It is also important to train health professionals in time management and counselling skills to equip them to adequately support postpartum women. Including these components in the design while tailoring interventions to postpartum women’s specific needs and addressing barriers may be effective strategies to improve penetration and participation and reduce attrition rate [91,94]. Considering that women prioritize their child’s wellbeing over theirs [84], involving the whole family in lifestyle interventions may be most effective as it consolidates partner support with women’s desire to be role models for their children [84,111]. One strategy to do this is to co-design lifestyle interventions and include postpartum women and their partner in the planning, development and implementation processes [91,111]. This will ensure that postpartum interventions reach the target population and are adequately engaged with, resulting in sustained impact of these interventions. This is necessary to improve implementation by improving program feasibility for adoption and acceptability among women leading to increased program effectiveness.

Considering the high prevalence of postpartum weight retention and the lack of translation of research evidence to practice on this topic, this review is an imperative for action to address implementation challenges through pragmatic, real-world trials for the effective translation of evidence into clinical practice to reduce postpartum weight retention. Future research should address knowledge gaps in terms of identifying the optimal timing for the initiation of lifestyle interventions. Randomized controlled trials of lifestyle interventions need to adequately report penetration, participation and other intervention characteristics to enable translation and replication of trials into practice. There is need for more research into the capacity building of health professionals to provide support and for a review of clinical practice guidelines for postpartum care to include lifestyle counselling.

## Figures and Tables

**Table 1:** Summary of systematic reviews examining the predictors of postpartum weight retention.

Author, Year	Main Aim	Study Type/Population	Number of Studies Included	Meta-Analysis Performed	Key Findings	AMSTAR 2 Rating
Bijlholt et al., 2020 [39]	To examine the association between eating behaviours and peripartum weight change	Quantitative studies Pregnant and PP women (up to 1 year after birth)	20	No	**Eating behaviour** Higher PP weight loss associated with Higher restrained eating (2/4 studies) Intuitive eating (1/1 study)	MQ
Lee et al., 2020 [30]	To examine changes in women’s diets from pregnancy to PP and the characteristics of women making these changes	Observational studies Pregnant and PP women	17	No	**Diet** From pregnancy to postpartum: Mixed findings for changes in energy and micronutrient intakes Significant decreases in fruit and vegetable consumption, diet quality, and adherence to a healthier dietary pattern Increases in discretionary food and fat intake	LQ
Hartley et al., 2018 [40]	To review the evidence on associations between PP depressive symptoms, anxiety symptoms, body image and weight status in the first 12 months post birth	Primary studies Women up to 12 months PP	12	No	**Psychosocial** Significant relationship between depressive symptoms, body image or weight status No significant associations between anxiety symptoms, body image or weight Body dissatisfaction was associated significantly with poorer PP weight status	LQ
Nagl et al., 2015 [41]	To provide an overview of the current state of evidence concerning associations between ante- and postnatal anxiety and pregnancy obesity, excessive GWG and PPWR	Empirical studies Pregnancy and PP	13	No	**Psychosocial** Positive association of pregnancy obesity and ante- or postnatal anxiety Insufficient studies on associations between excessive GWG (2 studies) or PPWR (3 studies) and anxiety	MQ
Xiao et al., 2014 [42]	To review the impact of sleep, stress and/or depression on PPWR	Primary studies Women (up to 5 years PP)	13	No	**Sleep** Positive association between short sleep duration and PPWR **Psychosocial** Non-significant associations between PP stress and weight retention Positive associations between PND and PPWR	LQ
Milgrom et al., 2012 [43]	To review the literature reporting on the relationship between ante- and postnatal maternal depressive symptoms and both maternal and childhood obesity	Primary studies Women and children	14 9 (maternal obesity) 5 (child obesity)	No	**Psychosocial** Positive association between PND and maternal obesity	MQ
Jiang et al., 2018 [44]	To clarify the relationship between different BF duration and PPWR through meta-analysis	Primary studies PP women	14	Yes	**Breastfeeding** Significantly lower PPWR in BF mothers compared with bottle-feeding mothers [−0.38 kg (95%CI −0.64, −0.11 kg)] **Others** Mothers who were primipara, <30 years old or with normal pre-pregnancy BMI had lower PPWR	MQ
He et al., 2015 [45]	To investigate the relationship between BF and PPWR	RCTs and cohort studies PP women	11	No	**Breastfeeding** Negative influence of BF for 3 to ≤6 months on PPWR No influence if BF continued for >6 months	MQ
Neville et al., 2014 [46]	To appraise the literature on the impact of BF on PP weight change, PPWR and maternal body composition	Prospective and retrospective studies BF mothers ≤2 years PP with BMI > 18.5 kg/m^2^	45	No	**Breastfeeding** Majority reported little or no association between BF and change in weight or body composition However, 4/5 studies of high methodological quality reported positive association between BF and weight change	CLQ

RCTs—randomised controlled trials; PP—postpartum; PPWR—postpartum weight retention; GWG—gestational weight gain; PND—postpartum/postnatal depression; 95% CI—95% Confidence Interval; BMI—body mass index; BF—breastfeeding; CLQ—critically low quality; LQ—low quality; MQ—moderate quality.

**Table 2:** Summary of systematic reviews examining the efficacy of lifestyle interventions in postpartum women.

Author, Year	Main Aim	Study Type/Population	Number of Studies Included	Meta-Analysis Performed	Efficacy Findings	Implementation Findings	AMSTAR 2 Rating
Lim et al., 2020 [17]	To assess the penetration, implementation, participation and effect of RCTs of lifestyle interventions in PP women	RCTs PP women (within 2 years after birth)	49	Yes		**Population penetration rate**: 2.5% **Program fidelity**: low in >50% of studies **Participation rate**: 0.94% to 86%	MQ
Lim et al., 2020 [83]	To describe the associations between behavioural strategies and changes in weight, diet, and PA in PP women	RCTs PP women (within 2 years of delivery)	46	Yes	**Weight:** Significant decrease (MD = −2.46 kg; 95%CI, −3.65 to −1.27) **Physical activity:** Significant increase (standardized MD = 0.61; 95%CI, 0.20 to 1.02) **Energy intake:** No significant change	**Behavioural strategies:** Those relating to self-regulation associated with greater reduction in energy intake	MQ
Buelo et al., 2019 [84]	To explore the effectiveness of PA interventions for women with previous GDM	Quantitative and qualitative studies Women with previous GDM	28	No	**Physical activity** 4 interventions significantly increased PA and 14 had either mixed effectiveness or no changes in PA		MQ
Ferguson et al., 2019 [87]	To investigate the effectiveness of lifestyle weight management interventions for PP women	Systematic reviews of RCTs and/or quasi-RCTs PP women	9	Yes	**Weight** PA + diet intervention resulted in a reduction in PP weight (MD = −1.7 kg; 95% CI, −2.3 to −1.1)		MQ
Lim et al., 2019 [79]	To evaluate the intervention characteristics associated with weight loss in PP women using the Template for Intervention Description and Replication framework	RCTs PP women (within 2 years after birth)	33	Yes		**Delivery** Health professional-delivered interventions had greater weight loss **Intervention type** Diet and PA combined had significantly greater weight loss compared with PA only interventions	MQ
Vincze et al., 2019 [71]	To evaluate the effectiveness of interventions that include a nutrition component aimed at improving GWG and/or PPWR	Studies of weight management interventions with a nutrition component Women (≥18 years) during pregnancy and up to 12 months PP	48	No	**Weight** - 9/14 studies conducted PP reported lesser PPWR - PP interventions more effective at improving weight outcomes compared to usual care or other interventions		MQ
Farpour-Lambert et al., 2018 [76]	To identify effective lifestyle interventions to manage weight and improve maternal and infant outcomes during pregnancy and PP	Systematic reviews or meta-analyses of RCTs Pregnant or PP women	15	No	**Weight** Combined diet + PA interventions reduce PPWR in women of any BMI (weighted MD = −2.57 to −2.3 kg) or with overweight/ obesity (weighted MD = −3.6 to −1.22 kg)		MQ
Dalrymple et al., 2018 [16]	To evaluate the effectiveness of lifestyle interventions in overweight or obese pregnant and/or PP women for managing PP weight up to 2 years after birth	RCTs During pregnancy and up to 2 years PP	18	No	**Weight** Seven PP only interventions reported significant improvements in PP weight when compared to the control group One pregnancy only and one pregnancy and PP intervention reported reduced PPWR at 6 months		LQ
Dodd et al., 2018 [74]	To evaluate PP dietary and/or PA interventions to promote weight loss and improve health in a subsequent pregnancy	RCTs Women with overweight or obesity, excessive GWG or PPWR	27	Yes	**Weight** Combined diet and PA intervention produced greater PP weight loss (MD, 2.49 kg; 95%CI, 3.34 to 1.63 kg) maintained at 12 months PP compared with no intervention		MQ
Mertens et al., 2018 [88]	To explore the existing literature about the effect of technology-supported lifestyle interventions including telemonitoring and coaching on GWG and PPWR	Quantitative, RCTs, protocol of RCTs Prenatal or postnatal women without medical conditions	9	No	**Weight** Technology supported interventions can optimize PPWR **Physical activity and diet** Inconsistent effects on PA and healthy eating	**Intervention characteristics** Delivered via - website - mobile application - SMS - website + SMS All used self-monitoring	MQ
Saligheh et al., 2017 [58]	To determine the efficacy of exercise or PA interventions on postnatal depression and weight loss	Intervention studies PP women (1 week to 24 months) diagnosed with postnatal depression	9	No	**Postnatal depression and Weight** - Inconsistency on the efficacy of PA to simultaneously reduce PND symptoms and assist weight loss - Two studies identified changes in both outcomes with small effect sizes. - Four studies reported changes in one outcome, typically PND with variable effect sizes - Three studies reported no effect		MQ
Elliott-Sale et al., 2015 [75]	To review the evidence from studies employing exercise-only interventions for weight management among pregnant and PP women	RCTs or quasi-randomized trials Pregnant or PP women (12 months post birth)	5	No	**Weight** Exercise significantly reduced GWG and had no significant effect on PP weight loss or BMI during pregnancy or PP		LQ
Gilinsky et al., 2015 [85]	To report the efficacy of postnatal PA interventions to change PA and walking behaviour and explore BCTs associated with efficacious interventions	Intervention studies PP women within 1 year after childbirth	20	Yes	**PA behaviour** - Interventions in healthy women but not for weight management successfully changed PA (Standardized MD = 0.53; 95% CI 0.05, 1.01) - Increase in frequency but not volume of PA or walking behaviour	**BCTs** Efficacious interventions always included BCTs - goal setting (behaviour) - prompt self-monitoring of behaviour	MQ
Gilinsky et al., 2015 [89]	To review studies investigating lifestyle interventions for women with prior GDM to explore changes in diet, PA, sedentary behaviour, anthropometric outcomes, glycaemic control and diabetes risk	RCTs, controlled or pre-post design trials Women with previous GDM	13	Yes	**Weight** Significant weight loss attributed to one Chinese population study (MD −1.06 kg; 95% CI −1.68, −0.44) **Diet** Positive effects on dietary variables in 6/6 studies reporting on diet **PA behaviour** Increases in PA in 6/11 studies **Others** No change in - fasting blood glucose - type 2 diabetes risk		MQ
Lim et al., 2015 [90]	To identify intervention strategies associated with weight loss in PP women	Intervention studies Women in their first year PP	46	Yes		**Intervention type** Interventions using self-monitoring and combined diet and PA were more effective on weight loss compared with PA alone	LQ
Berger et al., 2014 [86]	To examine the effect of PP nutrition and exercise interventions on weight loss and metabolic outcomes	RCTs PP women	13	Yes	**Weight** - Greater weight loss in the combined intervention group vs. standard care (range 0.17 kg to 4.9 kg) - Inconclusive results from exercise only interventions - Insufficient evidence for nutrition only interventions		MQ
Nascimento et al., 2014 [80]	To evaluate the effectiveness of lifestyle modification control trials that utilize exercise interventions, with or without dietary intervention, on weight loss among PP women	RCTs PP women	11	Yes	**Weight** MD on weight loss = −2.57 kg (95% CI −3.66 to −1.47)	**Intervention type** Most effective were: - Exercise programs with objectively defined goals, such as the use of heart rate monitors or pedometer - Exercise combined with intensive dietary intervention	MQ
Neville et al., 2014 [81]	To review literature on the effectiveness of weight management interventions in breastfeeding women	Intervention studies Breastfeeding mothers only (≤2 years PP)	6	No	**Weight** Dietary-based intervention studies most efficacious in promoting weight loss	**Delivery** Few studies were tailored toward the needs of breastfeeding women	CLQ
Adegboye et al., 2013 [72]	To evaluate the effect of diet, exercise or both for weight reduction in women after childbirth	Published and unpublished RCTs and quasi-randomized trials PP women	14	Yes	**Weight** - No significant difference in weight loss in exercise-only group - Significantly more weight loss in women in diet or diet + exercise program - No difference in the magnitude of weight loss between diet alone and diet plus exercise group		HQ
Choi et al., 2013 [73]	To review the effectiveness of PA and PA plus diet interventions in managing weight among pregnant or PP women with overweight or obesity	RCTs PP women with overweight or obesity	11	Yes	**Weight** Significantly more weight loss (−1.22 kg; 95% CI: −1.89, −0.56) in intervention group	**Intervention type** Supervised PA plus diet interventions were the most effective	LQ
Van der Plight et al., 2013 [82]	To evaluate the effectiveness of lifestyle interventions aimed at reducing PPWR	Intervention studies PP women	11	No	**Weight** Seven studies successful in decreasing PPWR	**Intervention type** Six successful interventions included dietary + PA components **Delivery** via a range of methods and delivered by a variety of health practitioners	LQ
Hoedjes et al., 2010 [77]	To review the effectiveness of PP lifestyle interventions aimed at weight loss, smoking cessation and smoking relapse prevention	Original research PP women up to 1 year after delivery	21	No	**Weight** 6/8 weight loss interventions were effective	**Delivery** Individually tailored counselling, group counselling sessions and use of diaries or other correspondence materials were effective	CLQ
Keller et al., 2008 [91]	To identify the best evidence available for guiding weight management interventions in postpartum women	RCTs PP women	6	No	**Weight** Significant impact of diet and exercise or some combination on body composition		CLQ
Kuhlmann et al., 2008 [78]	To assess whether effective weight management interventions exist for pregnant or PP women	RCTs Pregnant or PP women	3 (2 PP, 1 pregnant)	No	**Weight** Less PPWR in intervention group than control group in the two PP studies	**Attrition rate**: high in all studies	MQ

RCTs—Randomized Controlled Trials; PP—Postpartum; PA—physical activity; PND—Postpartum/postnatal depression; MD—mean difference; 95%CI—95% confidence interval; GDM—gestational diabetes mellitus; DM—Diabetes Mellitus; PPWR—postpartum weight retention; GWG—gestational weight gain; BMI—body mass index; BCTs—Behaviour Change Techniques; CLQ—critically low quality; LQ—low quality; MQ—moderate quality; HQ—high quality.
